# An exceptional case of peritoneal cystic echinococcosis in a domestic cat in Australia

**DOI:** 10.1111/avj.13427

**Published:** 2025-02-17

**Authors:** S Abdullah, H Kang, S Goodwin, A Dadhich, D McGilp, J Thomson, MK Jones

**Affiliations:** ^1^ The University of Queensland School of Veterinary Science Gatton Queensland Australia; ^2^ Killarney Vets Killarney Queensland Australia

**Keywords:** cystic echinococcosis (CE), *Echinococcus granulosus*, feline, feline leukemia virus, FeLV, immunocompromised

## Abstract

A case of peritoneal cystic echinococcosis (CE) in a domestic cat was reported from Queensland, Australia. Physical examination of the cat indicated a large, distended abdomen, which when palpated appeared to be fluid‐filled. Ultrasonography showed numerous cysts with hyperechoic walls and anechoic contents within the cat's abdominal cavity. Whole mount microscopy and histology, together with molecular identification of the contents based on mitochondrial DNA gene sequencing indicated that the causative agent was *Echinococcus granulosus* (*sensu stricto*) (genotype G1). Moreover, the cat was also found infected with Feline Leukemia Virus (FeLV). It is surmised that FeLV‐induced immunosuppression could have led to the development of CE in this cat. This is the first report of CE in a FeLV infected cat in Australia.

The genus *Echinococcus* (Rudolphi, 1801) is a member of the family Taeniidae, an important genus of cestodes causing zoonoses worldwide. As with other members of the family Taeniidae, the life cycle of *Echinococcus* species involves two mammalian hosts. Definitive hosts of *Echinococcus* spp. are canids and felids. These hosts carry the hermaphroditic adult tapeworms in their small intestine, releasing egg‐laden proglottids in the environment with their faeces. Intermediate hosts of *Echinococcus* species are usually ungulates and rodents, which act as prey for the definitive hosts. The infection of intermediate hosts is a result of ingestion of parasite eggs shed in the faeces of definitive hosts, and the definitive host is infected by ingesting intermediate hosts harbouring *Echinococcus* spp. metacestodes.^1^, ^2^

Based on morphological, molecular and ecological criteria, genus *Echinococcus* is now split into 10 species^3^; however, so far only *Echinococcus granulosus sensu stricto* (*s.s*) has been reported in Australia. In Australia, *E. granulosus* (*s.s*) genotype G1 is currently the only *Echinococcus* taxon present, transmitted between domestic dogs and sheep, comprising the domestic life cycle. The parasite is also widespread in wildlife in Australia, and its life cycle is now predominantly sustained through a sylvatic mode of transmission, which mostly involves dingoes (*Canis lupus dingo*), dingo‐ dog hybrids (*Canis lupus dingo* × *Canis familiaris*) and red foxes (*Vulpes vulpes*) as definitive hosts.^4^, ^5^, ^6^, ^7^ In the sylvatic cycle, macropodids (Family Macropodidae) and feral pigs (*Sus scrofa*) act as intermediate hosts.^5^, ^8^, ^9^ The sylvatic hosts are also reservoirs for infection of domestic species and humans.^10^, ^11^

Although cats are ubiquitous components of urban and rural landscapes in Australia and have access to macropods as food, either by predation on smaller species or eating of animals killed by wild dogs or road accidents, larval form of *E. granulosus* infection has not been reported in Australian domestic or feral cats. It is generally accepted that feral cats in Australia play no part in the transmission of this parasite.^5^, ^12^ However, in other parts of the world, *Echinococcus felidis* in Africa^13^ and *Echinococcus oligarthrus* in South America have wild felids as the main definitive hosts.^14^ Furthermore, *E. multilocularis* can develop in domestic cats, albeit with very low egg numbers in gravid proglottids.^15^

Felids in general are refractory to infection and survival of *E. granulosus s.s* adult stages. However, cystic echinococcosis (CE) has been reported in the past in New Zealand,^16^, ^17^ Europe^18^ and South America.^19^ More recent reports of infections of cats with larval form of *E. granulosus s.s*. emerged from Turkey,^20^, ^21^ Russia,^22^ Uruguay^23^ and Italy.^24^ A recent report^24^ indicates that CE is an opportunistic infection and develops mostly in immunocompromised cats. Those authors found the cat with CE was positive for feline immunodeficiency virus (FIV) infection. The virus‐induced, immunodeficiency is proposed to have led to opportunistic infection with *E. granulosus* metacestodes.

Feline leukemia virus (FeLV) and feline immunodeficiency virus (FIV) are retroviruses with a global impact on the health of domestic cats. Belonging to the family *Retroviridae*, the two viruses differ in their potential to cause disease. FIV can cause an acquired immunodeficiency syndrome that increases the risk of developing opportunistic infections, neurological diseases and tumours. In most naturally infected cats, however, FIV itself does not cause severe clinical signs, and FIV‐infected cats may live many years without any health problems.^25^, ^26^ FeLV on the other hand is more pathogenic and can cause tumours, bone marrow suppression syndromes and lead to secondary infections caused by suppressive effects of the virus on bone marrow and the immune system. Life expectancy of an FeLV infected cat ranges between 2 to 3 years.^25^, ^27^

The present report describes an exceptional case of an FeLV positive domestic cat infected with metacestode stages of *E. granulosus* G1 genotype, confirmed by molecular analysis. In keeping with recommended nomenclature for stages of the *E. granulosus* life cycle, the term ‘hydatid cyst’ will not be used herein, being replaced with the term ‘CE cyst’ or, more simply, ‘cyst’.^28^

## Methods and results

### Patient history and physical examination

A three‐year‐old female, spayed, Domestic Shorthair cat was initially examined on 14 of March 2023 by a veterinarian at the owner's property on a large farm in the Southern Darling Downs, Queensland, Australia. The owner had noticed a distention in their cat's abdomen over the past few weeks. The cat was allowed to roam freely around the farm buildings and had a history of foraging, often eating bush rats. The owner reported that the cat was eating, drinking, urinating and defecating normally and was up to date with all prophylactic treatments for parasites. Physical examination of the cat revealed a distended abdomen, that palpated as fluid‐filled, but the cat was otherwise unremarkable. Blood was taken from the right jugular vein for in‐house complete blood count and biochemistry. The only abnormalities detected were mild monocytosis and eosinophilia. The presumptive diagnosis was high parasitic burden causing ascites and with the history of foraging it was recommended to increase the deworming schedule to bi‐weekly, rather than every 3 months.

**Figure 1:**
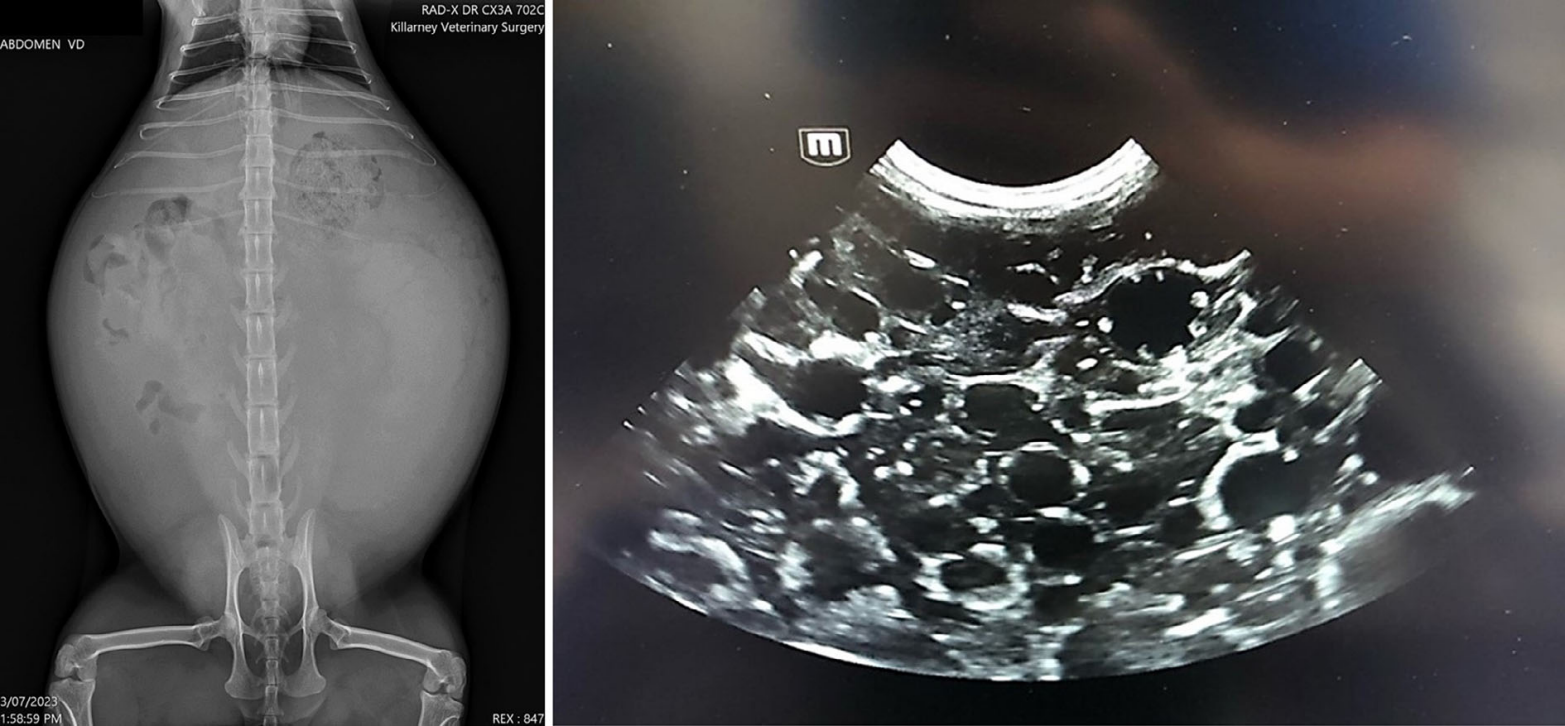
Radiograph of abdomen of the cat revealing soft tissue/fluid opacity taking up approximately 80% of the abdominal cavity (A). Abdominal ultrasound images (B) showing numerous, well demarcated, round structures of varying size (5–30 mm) with anechoic centres with thin (1–2 mm), hyperechoic walls.

After almost 3 months, on 4 of July 2023, the cat was brought to the clinic due to a remarkable increase in size of her abdomen. The physical examination again revealed the large, distended abdomen, that had almost doubled in size. Radiographs (represented in Figure 1A) revealed soft tissue/fluid opacity taking up approximately 80% of the abdominal cavity with some intestinal tract visible dorsally at the retroperitoneal space and a large ingesta‐filled stomach cranially. Ultrasound examination of the abdomen revealed numerous, well demarcated, spherical structures of varying size (5–30 mm) with anechoic centres with thin (1‐2 mm) hyperechoic walls (Figure 1B). Based on the diagnostic imaging results, exploratory laparotomy was elected for this cat.

### Exploratory laparotomy

**Figure 2:**
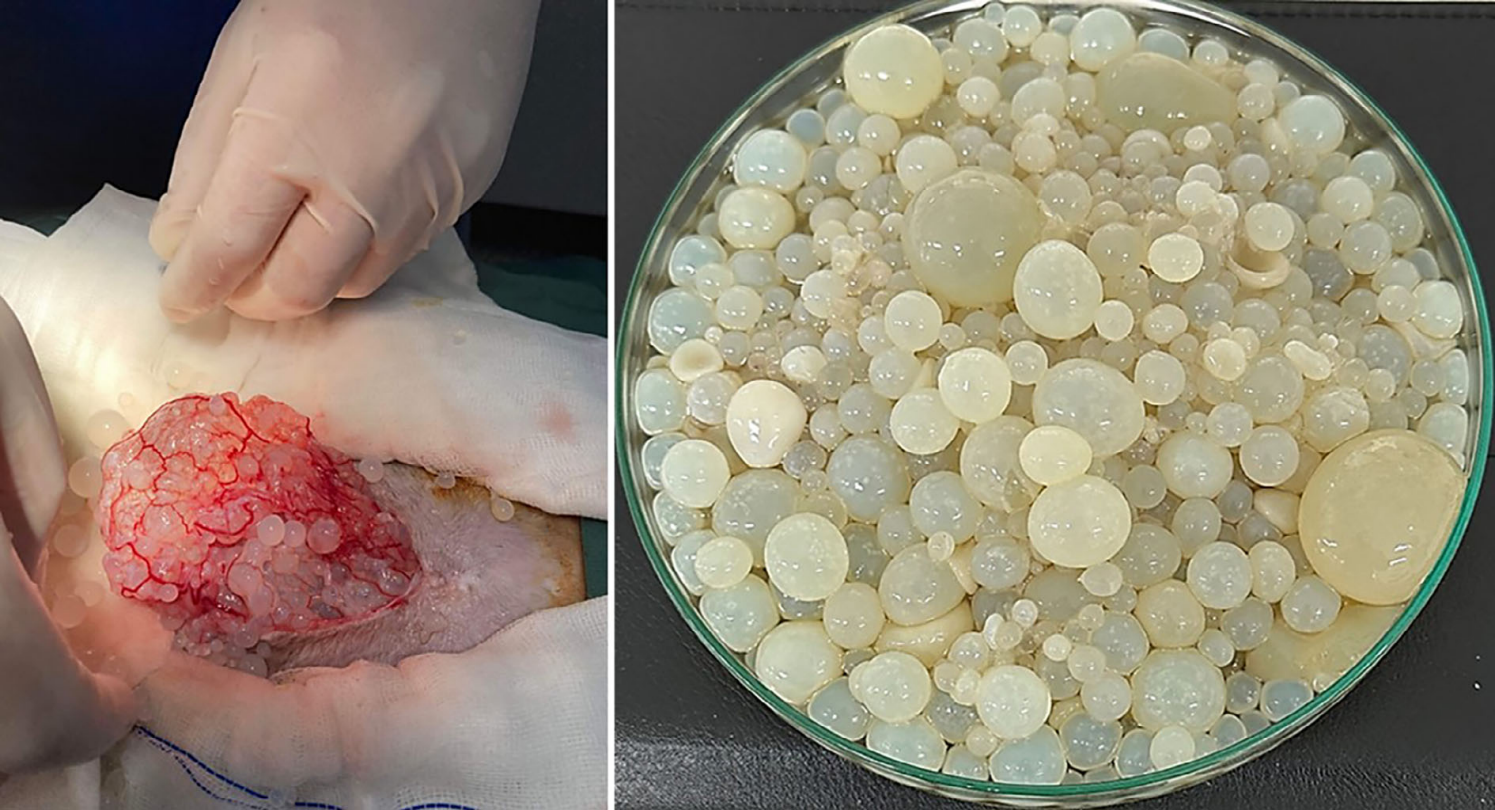
*Echinococcus granulosus* cysts collected from cat abdomen during exploratory laparotomy.

**Figure 3:**
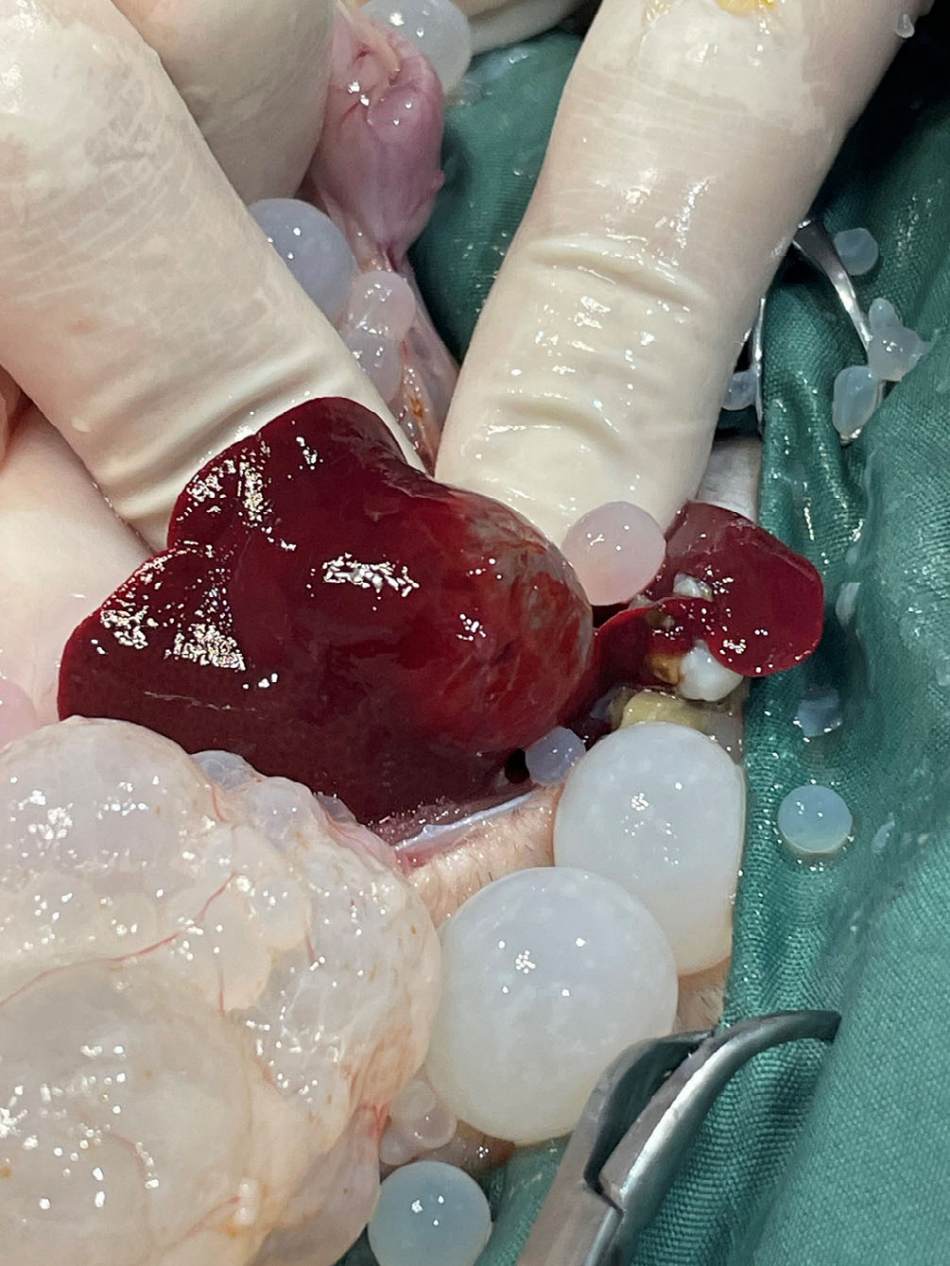
*Echinococcus granulosus* cyst embedded in liver parenchyma identified during exploratory laparotomy.

Exploratory laparotomy was conducted on 6 of March 2023. The cat was sedated with 0.3 mg/kg methadone (10 mg/mL) and 0.02 mg/kg acepromazine (2 mg/mL) intramuscularly. A 22G catheter was placed in the right cephalic vein for induction of anaesthesia and intravenous fluid therapy. Anaesthesia was induced with alfaxalone 10 mg/mL titrated to effect and maintained with isoflurane via tracheal intubation. The cat was placed in dorsal recumbency, and with aseptic preparation of ventral abdomen with chlorhexidine, alcohol and iodine. A 4 cm midline incision was made through the skin, blunt dissection through subcutaneous layer and a stab incision made through linea alba extended with Metzenbaum scissors. Copious free cystic structures were evacuated from the abdomen. The cysts were found through the entire omentum (Figure 2) and a large intrahepatic lesion was noticed, consistent with a primary cyst (Figure 3). At this stage the owner was called and updated about the findings. After discussion with the owner, humane euthanasia was performed with 3 mL phenobarbitone sodium 325 mg/mL intravenously. Tissue and cyst samples were collected in formalin for histopathology and some cyst samples were preserved in 70% ethanol for molecular analysis. The samples were sent to the School of Veterinary Sciences, the University of Queensland.

### Morphological examination

**Figure 4:**
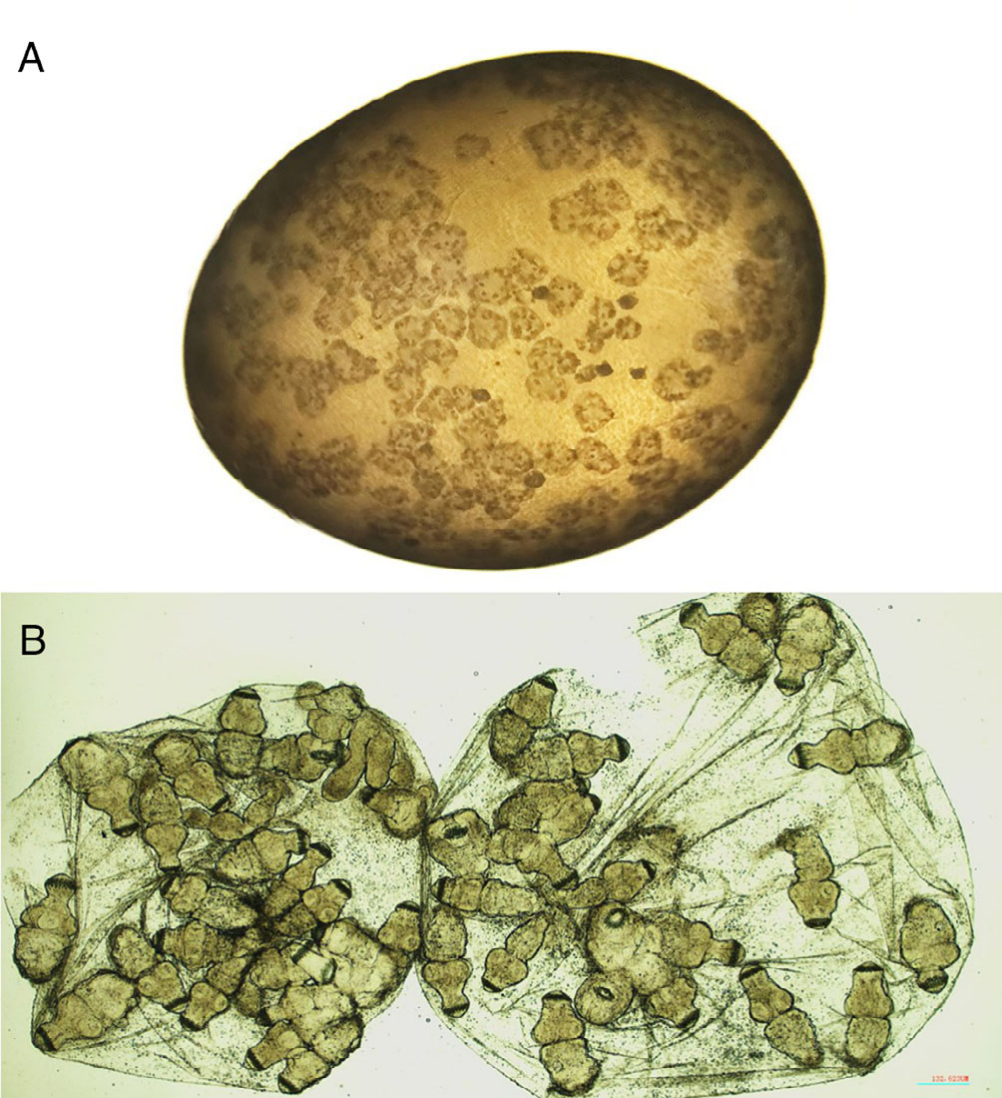
(A) *Echinococcus granulosus* cyst with brood capsules inside. (B) Brood capsules with everted protoscoleces.

Macroscopically, the peritoneal secondary cysts were fluid‐filled, spherical and translucent; and the brood capsules were easily recognised (Figure 4A). The diameter of the cysts ranged from 0.25 to 3.43 cm with an average diameter of 1.3 cm. Microscopically, the secondary cysts were composed of a germinal layer with brood capsules filled with everted protoscoleces (Figure 4B).

### Histopathology

**Figure 5:**
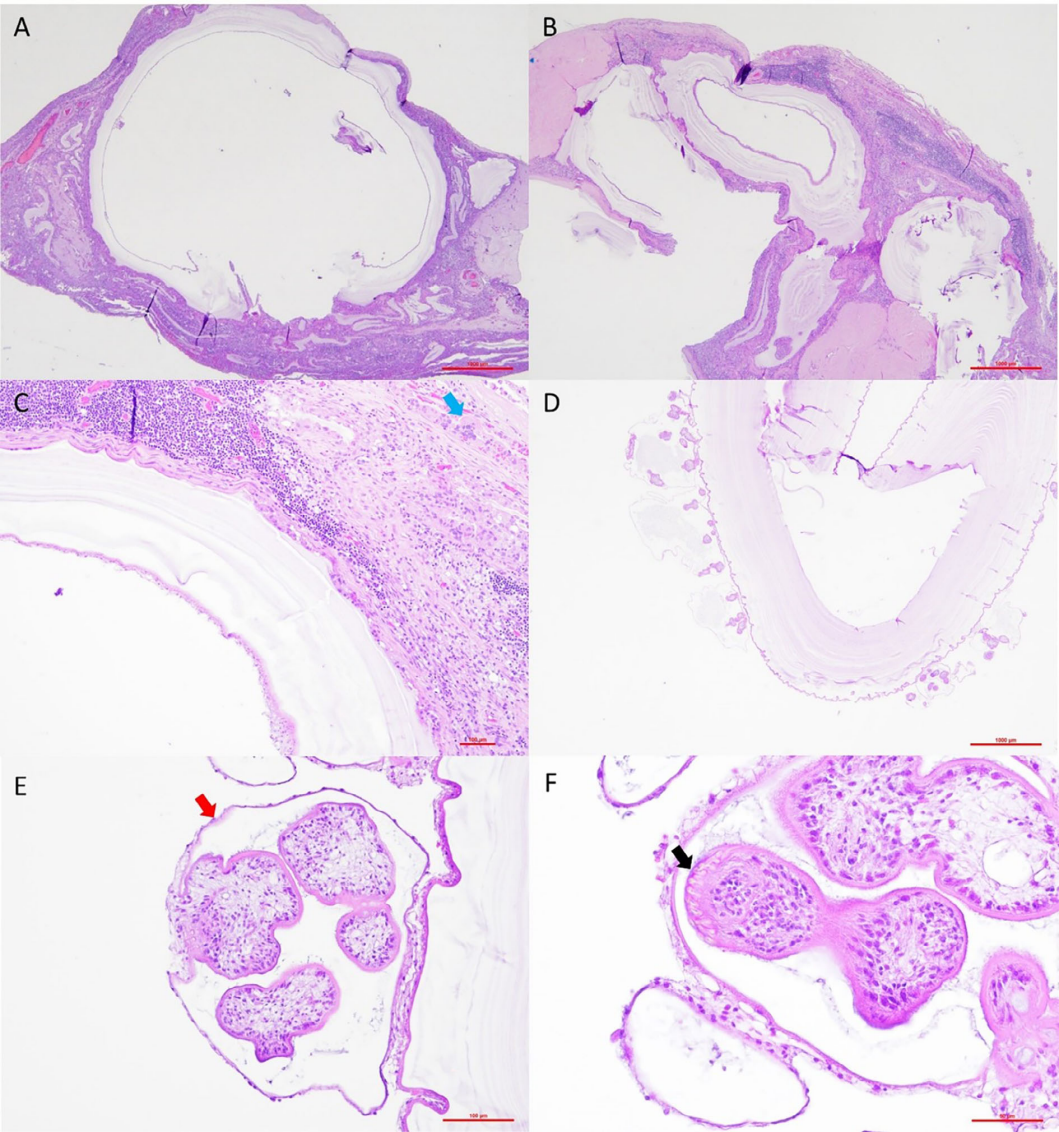
Histopathological features of *Echinococcus granulosus* cysts stained using haematoxylin and eosin. A single large, central, unilocular cyst with a pale eosinophilic‐hyalinised laminated wall surrounded by inflamed mesentery embedded with several serpentine cyst wall fragments from ruptured cysts, 20× magnification (A). Multiple unilocular cysts embedded in inflamed mesentery, 20× magnification (B). A higher magnification view of cyst with a germinal epithelium lining the clear space and laminated cyst wall surrounded by a large cluster of lymphocytes and plasma cells to the top left of the image as well as multinucleated giant cells (blue arrow), histiocytes and fibroblasts, 100× magnification (C). A single cyst with dozens of budding protoscoleces developing within brood capsules, 20× magnification (D). A single brood capsule (red arrow) budding from the cyst germinal epithelium and containing four profiles of protoscoleces, 200× magnification (E). A brood capsule with three profiles of protoscoleces, the hooks of the rostellum are evident (black arrow), in a single protoscolex the primary lacuna is evident, 400× magnification (F).

Formalin fixed cysts and peritoneum samples were subjected to histopathological examination. Sections were cut serially from paraffin blocks at 4 μm and stained using haematoxylin and eosin (H&E). Histopathological examination revealed multifocal and frequently embedded cysts within the peritoneum (Figure 5). The cysts were up to 10 mm diameter with round to ovoid clear spaces lined by a thin 10 μm simple germinal epithelial layer (the germinal membrane), occasionally containing basophilic, ovoid globular bodies (calcareous corpuscles). The germinal membrane was surrounded externally by an outer hyaline wall composed of lightly basophilic laminated layers (up to 800 μm thick), separated by a clear space. The germinal membrane had frequent invaginations that extend into the cyst lumen, the brood capsules. The germinal membranes of the capsules encircle dozens of 100‐150 μm diameter multi‐celled parasite profiles (the protoscoleces). Protoscoleces had a thin 5 μm eosinophilic tegument and a solid parenchymatous body occasionally attached to the brood capsule lining. Many protoscoleces were evaginated and displayed clear to light golden brown, birefringent hooks. Frequently concentrically surrounding cysts and further expanding the peritoneum were concentric, laminated layers of parallel, eosinophilic, fibrillar matrix embedded with numerous fibroblasts (fibrosis) surrounded and embedded with expanded epithelioid macrophages and multinucleated giant cells as well as large, dense clusters of lymphocytes and plasma cells and occasionally multifocal aggregates of extravasated erythrocytes (haemorrhage). Mesothelial cells at the serosal surface were up to sixfold expanded with moderate anisokaryosis and frequent multinucleation (reactive mesothelium). Multifocally and occasionally embedded within the peritoneum and surrounded by dense clusters of epithelioid macrophages, multinucleated giant cells, lymphocytes and plasma cells were fragments of laminated, hyalinised, lightly basophilic cyst wall (ruptured cysts). Free cysts lacked surrounding fibrosis and granulomatous inflammation noted within the host tissues that surround the mesentery embedded cysts.

### Molecular analysis

Cysts in 70% ethanol along with formalin fixed liver and spleen samples were sent to the University of Adelaide, School of Animal and Veterinary Sciences for molecular identification of the cysts and detection of possible FIV and FeLV infection. The DNA was extracted from cyst samples using RecoverAll™ Total Nucleic Acid Isolation Kit for FFPE. The extracted DNA was tested in specific PCRs for *E. granulosus*, *Echinococcus multilocularis* and *Echinococcus canadensis* in separate reactions. The PCRs showed positive results in *E. granulosus* and negative results for *Echinococcus multilocularis* and *Echinococcus canadensis*.^29^ Sequencing the positive PCR product showed the *E. granulosus s.s* genotype G1 with 99.36 identity to Australian isolates.

**Figure 6:**
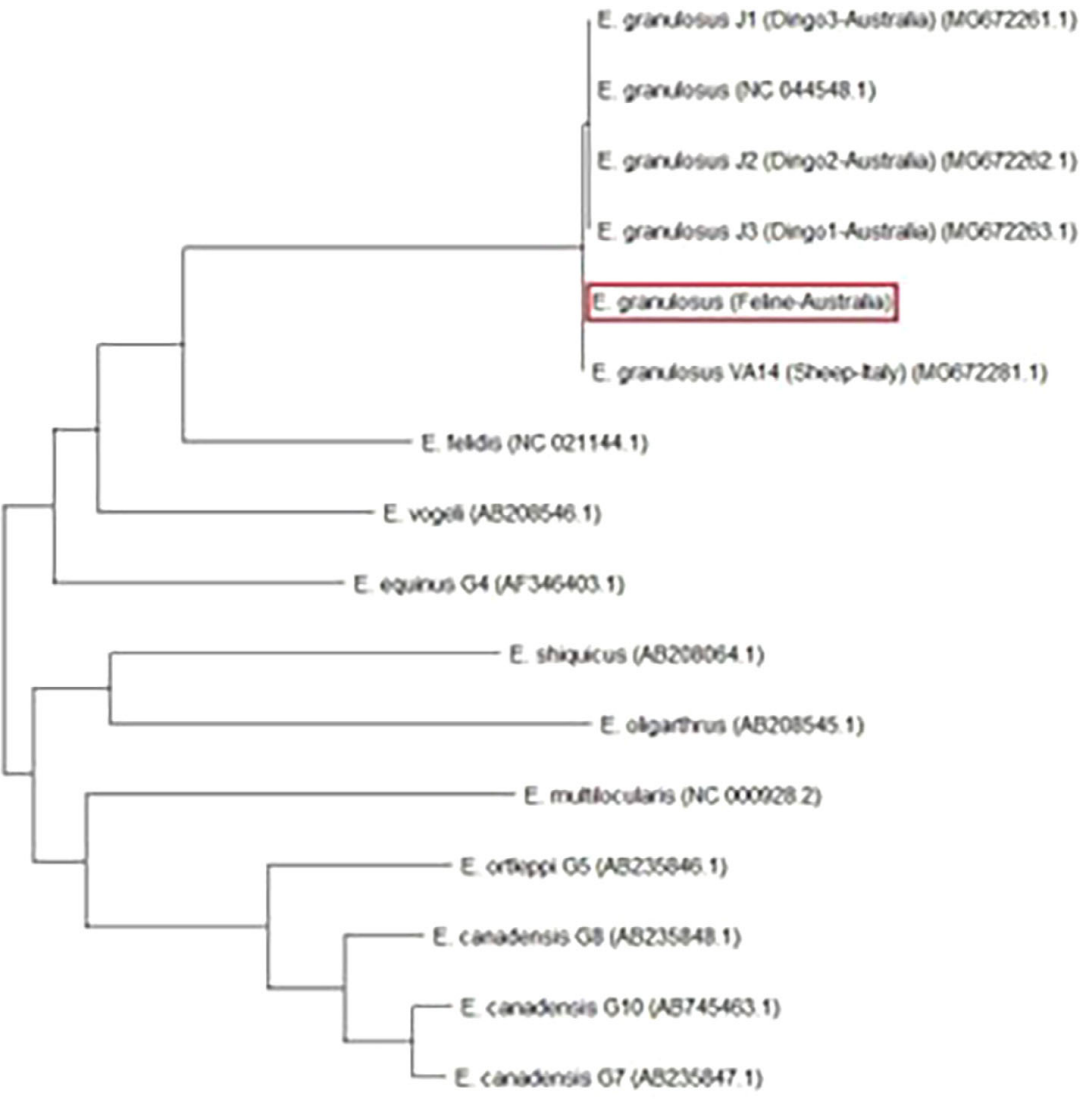
Phylogenetic tree constructed by the ‘maximum likelihood’ method based on mitochondrial adenosine triphosphatase subunit 6 (MT‐ATP6) gene. The ATP6 gene sequence of the current feline case (red box) was compared with ATP6 gene sequence of eight other *Echinococcus* spp., including *Echinococcus granulosus* infecting dingoes in Australia.

Approximately 740 bp of the mitochondrial adenosine triphosphatase subunit 6 (MT‐ATP6) gene area was used for phylogenetic analysis. A phylogenetic tree (Figure 6) was constructed using the ‘maximum likelihood’ method in MEGA 11.0.^30^ The gene sequences used for phylogenetic analysis included a reference sequence of *E. granulosus* (NC 044548.1), along with *E. granulosus* found in three dingoes in Australia (MG 672261.1, MG 672262.1 and MG 672263.1), and a sheep in Italy (MG 672281.1). We also included gene sequences from eight other *Echinococcus* species: *E. felidis* (NC 021144.1), *E. vogeli* (AB 208546.1), *E. equinus* Genotype 4 (AF 346403.1). *E. shiquicus* (AB 208064.1), *E. oligarthrus* (AB 208545.1), *E. multilocularis* (NC 000928.2), *E. ortleppi* Genotype 5 (AB 235846.1), *E. canadensis* Genotype 7 (AB 235847.1), Genotype 8 (AB 235848.1) and Genotype 10 (AB 745463.1). The sequence recovered from the domestic cat in this study was most closely aligned to *E. granulosus* VA14 and belonged to the same cluster as other *E. granulosus* sequences.

The RNA was extracted from formalin fixed liver and spleen samples using RecoverAll™ Total Nucleic Acid Isolation Kit for FFPE. The extracted RNAs were tested in nested PCRS for FIV and FeLV.^31^ The RNA from a positive FIV and FeLV samples were used as positive control nested one step RT‐PCR. Both liver and spleen samples showed positive results in nested PCRs for FeLV and negative for FIV.

## Discussion

This report on peritoneal CE in a domestic cat is the first for Australia. Cats do not harbour adult *E. granulosus* and are not normally an intermediate host for *E. granulosus*, and thus the incidence of CE in cats is extremely low. CE has been previously reported for cats in other countries.^16^, ^18^, ^19^, ^20^, ^21^, ^22^, ^23^, ^24^ Experimental infection of healthy domestic cats with *E. granulosus sensu lato* eggs has been unsuccessful.^32^ By contrast, a case report from Uruguay^23^ demonstrated CE in a cat that was co‐infected with FIV. In the present report, we show the presence of CE in a cat that is coincidentally FeLV positive. Both FIV and FeLV cause immunosuppressive disease in cats,^33^ and it can be suggested that CE mostly develops in cats that are immunocompromised.

The developmental origins of multiple cysts in the abdominal cavity of cats remain uncertain, although a possible explanation may be postulated. Most intermediate hosts of CE are infected by one parasite that becomes a single CE cyst. In most life cycles, the post‐embryonic larva, the oncosphere, hatches in the duodenum and penetrates the gut lining, eventually entering a lymphatic or blood vessel. From there, the oncosphere is carried to (usually) a solid organ in which it develops to form the cyst.^34^ In eutherian mammals, the primary site of cyst formation is the liver. Secondary peritoneal CE cysts can form after rupture of a primary live hepatic cyst.^35^ Primary cysts or any scars from these cysts in cats with cystic echinococcosis are not always detected,^16^, ^18^ however, in this case an entire cyst was found embedded in the liver parenchyma with no evidence of rupture. It remains uncertain whether the cyst in the liver was the primary cyst that led to development of secondary peritoneal cysts due to leakage of protoscoleces. In one study, some 16% of human patients with primary hepatic cysts also had secondary peritoneal cysts, suggesting that rupture or leakage of primary cysts is common.^36^

Although reports of CE in cats are rare, this current study and previous reports suggest that cats can be susceptible to infection with *E. granulosus* oncospheres, when they have immunosuppressive comorbidities. Infection of this cat serves as a reminder that the *E. granulosus* life cycle persists in the habitat of the cat, and that humans living in the area must remain vigilant against infection.^37^

This is another record that implicates immune suppression as a means that allows CE cysts to survive and thrive in what is an otherwise refractory host. Host physiological state is reported to influence the innate susceptibility or resistance to this infection and there are several reports of CE cysts in human patients linked to immunodeficiency disease.^38^, ^39^, ^40^, ^41^ This observation serves as a salutary reminder of the importance of host immune status on the ability of CE cysts to grow. In Australia, hosts such as cattle or pigs carry infertile CE cysts. In lacking protoscoleces, cysts of these hosts do not contribute to the life cycle. Selective breeding that indirectly influences some aspects of the immune status of the hosts might alter the ability of CE cysts to develop in such hosts.

## Conclusion

The findings of this report reiterate that CE can affect felines and should be included in differential diagnoses for abdominal distension in cats with evidence of multiple peritoneal cysts in ultrasound. Cats are not the natural intermediate host for this parasite, and this infection in cats is considered to be associated with immunosuppression which may favour the development of the metacestode stages. Therefore, it is important to check the immune status of these feline patients and test for FIV and FeLV infection in such situations.

## Conflicts of interest and sources of funding

The authors declare no conflicts of interest or sources of funding for the work presented here.

## Data Availability

Data sharing is not applicable to this article as no new data were created or analyzed in this study.
